# Supporting the advancement of science: Open access publishing and the role of mandates

**DOI:** 10.1186/1479-5876-10-13

**Published:** 2012-01-24

**Authors:** Lisa Phelps, Bernard A Fox, Francesco M Marincola

**Affiliations:** 1 BioMed Central, 236 Gray's Inn Road, Floor 6, London, WC1X 8HB, UK; 2 Earle A. Chiles Research Institute, Robert W. Franz Research Center, Providence Cancer Center, Providence Portland Medical Center, Portland, OR, USA; 3 Department of Molecular Microbiology and Immunology and Knight Cancer Institute, Oregon Health and Science University, Portland, OR, USA; 4 National Institutes of Health, Bethesda, Maryland, USA

## Abstract

In December 2011 the United States House of Representatives introduced a new bill, the Research Works Act (H.R.3699), which if passed could threaten the public's access to US government funded research. In a digital age when professional and lay parties alike look more and more to the online environment to keep up to date with developments in their fields, does this bill serve the best interests of the community? Those in support of the Research Works Act argue that government open access mandates undermine peer-review and take intellectual property from publishers without compensation, however journals like *Journal of Translational Medicine *show that this is not the case. *Journal of Translational Medicine *in affiliation with the Society for Immunotherapy of Cancer demonstrates how private and public organisations can work together for the advancement of science.

## Editorial

*Journal of Translational Medicine *is an open access journal published by BioMed Central that aims to optimise communication between basic and clinical science. Now in its 10^th ^year of publication the journal is successful in its aim for fostering communication from bench to bedside.

A new bill, the Research Works Act [1], has been introduced in the United States House of Representatives threatening the public's access to US government funded research and the foundation on which *Journal of Translational Medicine *was built. The bill states:

"No Federal agency may adopt, implement, maintain, continue, or otherwise engage in any policy, program, or other activity that:

(1) causes, permits, or authorizes network dissemination of any private-sector research work without the prior consent of the publisher of such work; or

(2) requires that any actual or prospective author, or the employer of such an actual or prospective author, assent to network dissemination of a private-sector research work."

If passed, this bill would force the retraction of the public access policy of the National Institutes of Health [2], who mandate that recipients of their grants must make their published research publically accessible by depositing full-text versions in open access repositories (such as PubMed Central), and prevent similar policies from being introduced by federal agencies in the future.

It is argued [3,4] that research funded by tax-payers should be made available to the public free of charge so that the tax-payer does not in effect pay twice for the research - first for the research to be done and then to read the results. As much as this may be true, the biggest detriment seems to be to developments in science. Open access to research means the widest possible dissemination of information. Limiting access to a (by comparison) small subset of people with subscriptions can stunt further developments.

When peer-reviewed information and data is made freely available online, text and data mining can help speed up advances by analysing and trending multiple datasets that would previously have taken years to achieve. As more information is made available, such investigations become more accurate and more meaningful.

Health care providers regularly access online resources including research to keep up to date with developments or to assist specific patients [5]. The same research also showed that health professionals would like greater access to information, including research. Access to the latest research is important for health care providers who may not always be attached to institutions or organisations with many journal subscriptions. Ensuring that clinicians and health care providers are aware of developments help move ideas from bench to bedside and can allow improved patient care by benefitting from the experience of others.

A recent commentary article [6] published in *Journal of Translational Medicine *discusses outcomes from a summit called by the Society for Immunotherapy of Cancer (SITC), which included representatives from both public and privately funded organizations from across the globe. They discuss various hurdles that hinder the progression of promising cancer immunotherapies to clinical application, although these hurdles may well resonate within other areas of translational medicine.

One hurdle discussed relates to the exchange of information between different groups of people stating: "...it is becoming less feasible for a single group to have the detailed knowledge and resources to investigate, analyze, select and implement the best strategies to move forward in clinical trials for any given indication."[6] This highlights the importance and continual need for sharing information and findings in an open way, to ensure that potential new therapies can continue to move forwards.

The SITC is affiliated with the "Tumor immunology and biological cancer therapy" section of *Journal of Translational Medicine*. They are one of a growing number of societies affiliated with or publishing open access journals in their fields. There are currently believed to be over 530 societies publishing at least one open access journal [7], a figure that is growing in accuracy thanks to the community editable record [8]. Maybe one of the reasons that open access is an increasingly popular choice for society journals is that it fits well with many society missions to encourage the advancement of knowledge by providing the widest possible dissemination with no barriers to access. While the Research Works Act is supported by the Association of American Publishers(AAP)[9], several of the AAPs 50+ society and publisher members have already distanced themselves from the AAPs support of the bill, including the American Association for the Advancement of Science, publisher of *Science*[10], and Nature Publishing Group [11].

Belief in the benefits of open access publishing continues to grow demonstrated by a reported 47% growth in open access articles published in 2011 compared to 2010 and 24% growth in the number of journals in the Directory of Open Access Journals [12]. Over 50 funding bodies worldwide currently have policies in support of open access [13] but by no means do these policies act to exclude publishers from continuing to publish articles compiled based on research funded by government bodies or grants. Publishers, regardless of the model by which they operate (subscription or open access covered by author charges), do need to cover the costs associated with the services they provide. Publishers who are traditionally recompensed for their services via subscription fees can (and do) offer open access options, allowing research to be deposited in public repositories such as PubMed Central after a certain period of time.

While author choice is fundamental in the publication process, given the importance of wide access to all research, whether public and privately funded, should we stop funders from requiring the results of their funding be as visible and transparent as possible?

## Competing interests

Lisa Phelps works for BioMed Central, publisher of *Journal of Translational Medicine*.

The Society for Immunotherapy of Cancer (SITC) is affiliated with the "Tumor immunology and biological cancer therapy" section of *Journal of Translational Medicine*.
